# 单克隆免疫球蛋白筛查与临床应用中国专家共识（2025年版）

**DOI:** 10.3760/cma.j.cn121090-20250108-00016

**Published:** 2025-04

**Authors:** 中国医药教育协会血液学专业委员会 中国医师协会血液科医师分会骨髓瘤专家委员会 中华医学会血液学分会浆细胞疾病学组

## Abstract

单克隆免疫球蛋白（M蛋白）是克隆性B细胞或浆细胞分泌的同种免疫球蛋白或其片段（如轻链）。M蛋白相关疾病是一类以血和（或）尿中存在M蛋白为主要特征的疾病。M蛋白筛查有助于早期发现这类疾病。为提高M蛋白相关疾病的早期发现率，国内相关领域专家制定了本共识，系统介绍现有的M蛋白筛查方法及其临床应用范围，以指导临床实践。

## Abstract (en)

Monoclonal immunoglobulins（M proteins）are homogeneous immunoglobulins or their fragments（such as light chains）secreted by clonal B cells or plasma cells. M protein-related diseases are a group of disorders characterized by the presence of M proteins in the blood and/or urine. M protein screening helps in early M protein-related disease detection. To enhance the early detection of M protein-related diseases, domestic hematologists have developed this consensus, which systematically introduces the current screening methods for M proteins and their clinical application scenarios to guide clinical practice.

单克隆免疫球蛋白（M蛋白）由克隆性B细胞或浆细胞所产生，可以通过血液和（或）尿液检测发现，包括单克隆完整免疫球蛋白（immunoglobulin, Ig）（含重链和轻链）和（或）游离轻链（free light chain, FLC）[1]。M蛋白相关疾病包括相对常见的意义未明单克隆丙种球蛋白血症（MGUS），也包括相对罕见的孤立性浆细胞瘤（SPC）、冒烟型多发性骨髓瘤（SMM）、多发性骨髓瘤（MM）、浆细胞白血病（PCL）、华氏巨球蛋白血症（WM）、系统性轻链（AL）型淀粉样变、B细胞非霍奇金淋巴瘤（B-NHL）及其他B细胞淋巴增殖性疾病（LPD）等。这类疾病多见于老年人，随着中国人口老龄化加速，预计M蛋白相关疾病的发病人数将不断增加。

然而，M蛋白相关疾病的早期诊断在临床实践中仍面临较大挑战。以MM为例，患者在疾病早期往往无症状，在确诊时常已处于疾病较晚期。众所周知，早期发现疾病意义重大。每年约1％的MGUS人群会进展为症状性MM、其他浆细胞或B细胞恶性肿瘤[2]。几乎所有MM由MGUS进展而来，但只有不到10％的MM患者有明确的MGUS病史可追溯[3]–[4]。通过M蛋白筛查早期发现血和（或）尿中的M蛋白可以为基础疾病［如MM等恶性浆细胞或B细胞肿瘤、有临床意义的单克隆丙种球蛋白病（MGCS）及其他MGUS相关疾病］的早期诊断提供线索，对于改善患者的生存和预后具有重要的意义。

研究表明，主动筛查并确诊的MGUS患者较随机诊断患者有更少的合并症[5]。此外，MGUS不仅可能进展为恶性肿瘤，还可进展为MGCS。早期诊断和干预MGCS有助于改善患者症状，预防不可逆的器官损伤[6]。同时，MGUS与骨折、感染、神经病变、血栓形成、自身免疫性疾病及死亡率增加等相关[5]。

目前，国内M蛋白筛查比例较低，各单位检测手段不一。为提高医务人员对M蛋白相关疾病的诊断意识，规范不同临床场景下的筛查策略，实现基础疾病的早期诊断，中国医药教育协会血液学专业委员会、中国医师协会血液科医师分会骨髓瘤专家委员会及中华医学会血液学分会浆细胞学组联合制定了本共识。

一、M蛋白检测方法

血/尿蛋白电泳（serum/urine protein electrophoresis，SPE/UPE）、血/尿免疫固定电泳（serum/urine immunofixation electrophoresis，sIFE/uIFE）、毛细管电泳免疫分型（capillary electrophoresis immunotyping，IT）、血清游离轻链（serum free light chain，sFLC）检测、重轻链（heavy and light chain，HLC）检测以及质谱法（mass spectrometry，MS）均可用于检测M蛋白。每种方法各有相应的优势与局限性，临床实践中应按照各自的需求，选择上述方法中的一种或多种进行M蛋白筛查。

1. SPE/UPE：SPE/UPE是筛查M蛋白的最常用检测手段之一，可识别较高浓度的M蛋白并进行相对定量分析。该方法技术要求较低，有条件的单位均可开展。SPE/UPE的检测灵敏度较低，检测下限为0.3～0.5 g/L。目前，根据电泳介质不同，SPE/UPE可分为凝胶电泳和毛细管电泳。毛细管电泳法分辨率更高，且可实现全自动化操作。

2. sIFE/uIFE：IFE为定性分析，可对M蛋白进行具体分型，其检测灵敏度高于SPE/UPE。sIFE检测下限为0.2 g/L，uIFE灵敏度更高，检测下限可低至0.04 g/L。对于IFE显示为单纯轻链型的标本，应加做IgD和IgE的IFE检测，以排除少见的IgD或IgE型M蛋白。

3. IT：IT可对血清或尿液中的蛋白进行电泳，并确认M蛋白类型。IT采用毛细管内的缓冲液作为分离介质。与IFE相比，IT为全自动化操作，通过检测异常峰的消失和（或）减少确认M蛋白类型。在多克隆免疫球蛋白浓度较高的情况下，IT可识别微量的M蛋白。此外，IT的分辨率较IFE更佳。

4. sFLC检测：sFLC检测利用可特异性结合游离状态免疫球蛋白轻链靶表位的抗体对sFLC进行定量分析。sFLC检测灵敏度为0.01～0.03 g/L，可检测到微量sFLC。通过κ和λ FLC的比值（rFLC）判断克隆性。目前，sFLC检测主要采用免疫比浊法或酶联免疫吸附法。需要注意的是，对于不同sFLC检测方法，rFLC参考范围存在差异。肾功能不全患者rFLC的正常值高于肾功能正常人群。因此，建议根据估计的肾小球滤过率（eGFR）校正rFLC参考范围[7]。

5. HLC检测：HLC检测通过特异性识别免疫球蛋白重链与轻链恒定区的结合区域，对血清中同型重/轻链对（IgG κ/IgG λ，IgA κ/IgA λ，和IgM κ/IgM λ）进行定量分析，并根据HLC比值判断克隆性。

6. MS检测：目前，基质辅助激光解吸电离飞行时间质谱（MALDI-TOF MS）可通过血清完整轻链分析进行M蛋白检测。该方法具备高灵敏度、高通量和高自动化优势，主要包括两种样本处理方式：①血清直接还原后通过质谱检测血清总轻链并识别M蛋白[8]–[9]。该方式检测成本较低，灵敏度较高，可检出血清中约0.1 g/L的M蛋白，但无法进行M蛋白分型。②通过抗体免疫富集后经质谱对M蛋白进行分析[10]–[11]。虽然该方式检测成本较高，样本前处理耗时较长，但单个样本分析时间短（<30 s）。与IFE/IT相比，该方法检测灵敏度更高。

二、M蛋白筛查的临床应用

1. 疑诊为M蛋白相关疾病：怀疑存在M蛋白相关疾病时，及时进行M蛋白筛查对于基础疾病诊断至关重要。浆细胞或B细胞疾病，如MGUS、SPC、SMM、MM、PCL、WM、AL型淀粉样变、B-NHL、其他B细胞LPD及其他M蛋白相关疾病等，常伴M蛋白分泌[12]–[16]。M蛋白筛查不仅有助于这类疾病的早期诊断，还对疾病的疗效评估及预后分层具有重要意义。

MGCS是一组以血和（或）尿中存在M蛋白，并由M蛋白所致组织、器官损害为主要临床特征的疾病，多为少见或罕见疾病，容易被漏诊或误诊[17]。MGCS常见的疾病类型包括AL型淀粉样变[18]、具有肾脏意义的单克隆丙种球蛋白病（MGRS）[19]、具有神经意义的单克隆丙种球蛋白病[6]和具有皮肤意义的单克隆丙种球蛋白病[20]等。MGCS的临床表现多样，异质性强，可累及多个器官，包括心脏、肾脏、肝脏、消化道、自主或周围神经及皮肤组织等（表1）[17],[21]–[22]。因其非特异性的临床表现，患者首诊科室通常不是血液科，而是分布在心内科、肾内科、消化科、神经内科及皮肤科等其他科室。

**表1:** 不同类型有临床意义的单克隆丙种球蛋白病（MGCS）的主要特征和累及器官

疾病名称	主要特征	主要累及器官
系统性轻链型淀粉样变	λ LC（75％），κ LC（25％），IgM<10％	全身性（心脏80％，肾脏70％）
Ⅰ型冷球蛋白血症	IgG或IgM	全身性［皮肤为主，肾脏、周围神经或全身性症状（晶体冷球蛋白）］
Ⅱ型冷球蛋白血症	IgM	皮肤为主、肾脏、周围神经；也可能是全身性
免疫触须样肾小球病	类CLL克隆增殖（50％）	肾脏
范可尼综合征	κ LC占90％以上	肾脏（近端小管病变）
结晶贮存组织细胞增多症	κ LC	全身性（肾脏、角膜、关节、淋巴组织）
结晶性角膜炎	IgG	角膜
单克隆免疫球蛋白沉积病（MIDD）	LCDD：仅LC（通常为κ LC）；HCDD：仅HC（主要是γ1和γ3）；LHCDD：LC和HC	全身性［肾脏（约100％，肾小球和肾小管基底膜），肝脏（30％），心脏（30％）］
增生性肾小球肾炎伴单克隆免疫球蛋白沉积（PGNMID）	通常为IgG3	肾脏
巨球蛋白血症	IgM	皮肤（真皮）
C1抑制剂缺乏症	–	血管性水肿
血管性血友病（AvWD）	–	出血
大疱性皮肤病	–	皮肤
黄瘤病	通常为IgG	巨噬细胞中的胆固醇积累；皮肤和肌腱；其他位置（坏死性黄瘤病）
冷凝集素病（CAD）	IgM	冷诱导的皮肤表现和血管内溶血
IgM相关性周围神经病变（IgM-PN）	IgM	周围神经；共济失调性多发性神经病［抗髓鞘相关糖蛋白（MAG）抗体阳性］；CANOMAD综合征
C3肾小球肾炎	IgG	仅肾脏
非典型溶血性尿毒症综合征	–	全身性
POEMS综合征	λ LC（约100％），IgA占比约50％，骨硬化性病变	周围神经（100％）和其他各种表现
系统性毛细血管渗漏综合征	IgG、IgA（罕见）	全身性
TEMPI综合征	IgG	全身性
中性粒细胞皮肤病^a^	IgA占80％以上（Sweet综合征除外）	皮肤为主；不同类型的病变伴随不同表现
获得性皮肤松弛症	通常为IgG；与γ型HCDD相关	皮肤为主；其他表现（肺、消化道）
硬化性黏液水肿	电泳显示缓慢迁移的IgG	皮肤为主；其他部位
硬皮病	IgG	仅皮肤
Schnitzler综合征	IgM	皮肤为主；全身性症状；骨硬化性病变
散发迟发性杆状体肌病（SLONM）	–	仅限于肌肉（骨骼肌、也可能在心脏）

**注** LC：轻链；HC：重链；CLL：慢性淋巴细胞白血病；LCDD：轻链沉积病；HCDD：重链沉积病；LHCDD：轻链和重链沉积病；CANOMAD：慢性共济失调性神经病、眼肌麻痹、单克隆IgM蛋白、血冷凝集素及抗二酰基抗体阳性；POEMS：多发性神经病、器官肿大、内分泌病、单克隆免疫球蛋白、皮肤变化；TEMPI：血管瘤、红细胞增多症伴红细胞生成素水平正常、单克隆免疫球蛋白、肾周积液和肺内分流；^a^包括化脓性皮肤坏疽、Sweet综合征、皮下脓疱性皮炎和结节性红斑病；–：无数据

MGCS的早期诊断是提高患者生存率的关键，及时诊断和规范化治疗可改善患者临床结局。因此，当遇到不明原因的心肌病及心力衰竭、蛋白尿和（或）血尿、肾功能不全、肝脾肿大、周围神经麻木或疼痛、反复发作的慢性荨麻疹、不明原因的红细胞及血小板升高等情况时，临床医师应进行M蛋白筛查，以排查有无MGCS可能。

临床各相关科室若遇到下列情况者，建议筛查M蛋白。

（1）血液科：不明原因球蛋白升高或降低、贫血（特别是正细胞正色素性）、血小板升高、血常规淋巴细胞比例或绝对计数升高、外周血涂片显示红细胞缗钱样排列，以及肝脾淋巴结肿大。

（2）骨科：不明原因固定部位骨痛（特别是肋骨及脊柱）、溶骨性骨病变、不明原因骨折、与年龄不相符的骨质疏松（绝经前女性或65岁以下男性）、骨硬化。

（3）心内科：不明原因射血分数保留的心力衰竭、室壁增厚（特别是室间隔增厚）但心电图显示肢导低电压、不明原因N末端B型利钠肽前体（NT-proBNP）和（或）肌钙蛋白I/肌钙蛋白T（TNI/TNT）升高、体位性低血压、高血压患者血压突然正常。

（4）肾内科：不明原因蛋白尿（尿中泡沫增多）（尤其是白蛋白尿且不伴高血压）和（或）血尿（肾性血尿）、肾病综合征、尿常规蛋白阴性但24 h尿蛋白升高、血肌酐升高、肾周积液。

（5）风湿免疫科：不明原因雷诺现象、皮肤网状青斑、皮肤紫癜、皮肤溃疡及关节痛、伴或不伴溶血。

（6）神经内科：不明原因周围感觉和（或）运动神经异常（对称或不对称）、腕管综合征、眼睑下垂及共济失调。

（7）皮肤科：不明原因皮肤色素沉着、肾小球样血管瘤、多毛症、肢端发绀、白甲、毛细血管扩张、慢性荨麻疹、皮肤溃疡坏死、反复皮下紫癜（暗红色，不易消退）。

（8）内分泌科：不明原因性腺功能减退症和（或）高催乳素血症、肾上腺皮质功能减退症、甲状腺功能减退、高钙血症。

（9）呼吸科：不明原因肺间质病变、胸腔积液、肺内分流。

（10）消化内科：不明原因巨舌、肝脏肿大和（或）碱性磷酸酶升高、重度吸收不良伴腹泻和脂肪泻、慢性假性肠梗阻。

对于上述各科疑诊为M蛋白相关疾病的患者，应联合使用SPE、IFE/IT和sFLC检测进行M蛋白筛查【推荐程度：必须筛查】。

应注意，部分浆细胞或B细胞疾病患者使用上述方案筛查后，M蛋白检测结果可能仍为阴性，如非分泌型MM、WM、PCL、POEMS综合征及部分MGRS等。因此，还需联合其他手段排除相关疾病。

2. M蛋白相关疾病的高危人群：筛查目的是早期识别无症状M蛋白相关疾病高风险人群，使其可能获益于早期干预，从而改善整体生存率或生活质量。同时，基于风险与成本效益的权衡，对特定目标人群进行筛查具有重要的临床意义。

相对明确的危险因素包括但不限于年龄（60岁以上）、性别（男性）、种族（非洲裔）、M蛋白相关疾病患者的直系亲属、辐射及杀虫剂暴露史、患有炎症性或自身免疫性疾病者[23]。

对于上述高危人群（具有一个或多个危险因素者），应检测SPE和sFLC进行M蛋白筛查【推荐程度：建议筛查】。

3. 健康人群：在此共识中，不满足上述两类筛查情况且年龄在50岁以上者，被定义为健康人群。

M蛋白相关疾病中，MGUS的患病率最高。大部分MGUS无症状且可能终生不进展。即使少数患者最终进展，短期内进展概率较低。因此，针对健康人群的常规筛查可能带来潜在负面效应，包括不必要的心理负担和焦虑等[24]。

机会性筛查可能是一种更为理想的筛查策略。中国首个在医院人群中验证MM筛查效果的真实世界研究显示，在常规肝脏生化检查中加入SPE进行M蛋白筛查，1.95％的患者SPE结果呈阳性，7年内SPE阳性率维持在1.47％～2.33％[25]。与健康人群筛查相比，急诊或住院患者筛查的检出率更高，从卫生经济学角度来看，可能带来更为有利的结果[26]。

既往基于健康人群筛查的研究显示，50岁以上人群MGUS患病率为1％～4％，男性高于女性，且随年龄的增长逐渐升高[27]–[28]。来自北京协和医院的研究显示，50岁以上中国人群MGUS患病率为1.11％[29]。iStopMM是一项在冰岛进行的全国性筛查项目，旨在评估MGUS筛查的潜在危害与长期获益。这项大型前瞻性研究发现，40岁以上的冰岛居民中MGUS的患病率为4.9％[30]。随访3年后，强化随访组淋巴增殖性疾病的检出率显著升高，证实健康人群筛查可早期发现高肿瘤负荷疾病[31]。

中国人口基数庞大，老龄化趋势日益加剧，预计M蛋白相关疾病的绝对发病人数将逐年上升。而目前中国人群中M蛋白筛查率较低，多数患者确诊时已处于疾病较晚期。因此，开展M蛋白筛查对M蛋白相关疾病的早期诊断至关重要。SPE作为M蛋白检测的常规手段，具有操作简便、价格低廉等优势。通过对50岁以上健康人群进行SPE初筛，有望实现M蛋白相关疾病的早期诊断，为必要时的干预奠定基础，最终达到改善预后的目标。

建议对50岁以上健康人群进行SPE检测。若为阳性，则进一步完善sIFE和sFLC检测【推荐程度：建议有条件的中心开展筛查】。

合理的M蛋白筛查策略不仅有助于M蛋白相关疾病的早期诊断，还能在高危及健康人群中及时发现潜在患者，为后续全程管理奠定基础，改善患者预后。本共识结合国情，对不同情况下的M蛋白筛查策略作出推荐（图1），旨在为临床实践提供指导。

**图1:**
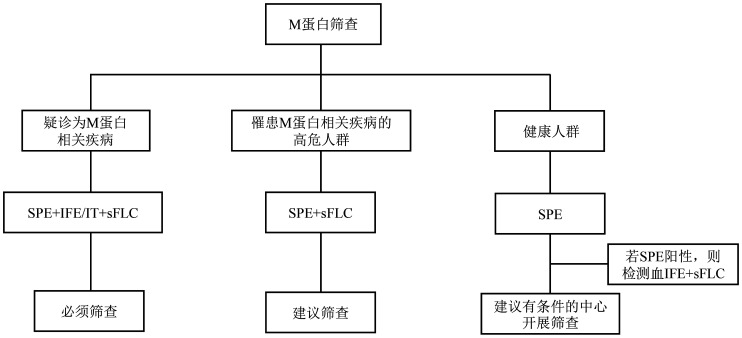
单克隆免疫球蛋白（M蛋白）筛查的临床应用 **注** SPE：血清蛋白电泳；IFE：免疫固定电泳；IT：毛细管电泳免疫分型；sFLC：血清游离轻链
